# Supplementary material for: Genome-wide Estrogen Receptor-α activation is sustained, not cyclical
Source: eLife. 2018 Nov 20;7:e40854. doi: 10.7554/eLife.40854 (PMC6287946; doi:10.7554/eLife.40854)
Supplement: Transparent reporting form [file elife-40854-transrepform.pdf]

eLIFE
1st Floor
24 Hills Road
Cambridge CB2 1JP, UK
P 01223 855340
W elifesciences.org
T @elife

eLife's transparent reporting form

We encourage authors to provide detailed information *within their submission* to facilitate the interpretation and replication of experiments. Authors can upload supporting documentation to indicate the use of appropriate reporting guidelines for health-related research (see EQUATOR Network), life science research (see the BioSharing Information Resource), or the ARRIVE guidelines for reporting work involving animal research. Where applicable, authors should refer to any relevant reporting standards documents in this form.

If you have any questions, please consult our Journal Policies and/or contact us: editorial@elifesciences.org.

Sample-size estimation

- You should state whether an appropriate sample size was computed when the study was being designed
- You should state the statistical method of sample size computation and any required assumptions
- If no explicit power analysis was used, you should describe how you decided what sample (replicate) size (number) to use

Please outline where this information can be found within the submission (e.g., sections or figure legends), or explain why this information doesn't apply to your submission:

No explicit power analysis was used. The sample size was chosen to maximize replication; however, this had to be balanced with cost and the level of sample processing that was achievable.

The total number of replicates (six) is much greater than most ChIP-seq studies (typically one or two). The number of time points (ten) is also significantly greater than the typical number of conditions (two or three). The study therefore acquired data at ten times the level of data collection that is typical for this work and did so for all conditions.

eLife Sciences Publications, Ltd is a limited liability non-profit non-stock corporation incorporated in the State of Delaware, USA, with company number 5030732, and is registered in the UK with company number FC030576 and branch number BR015634 at the address 1st Floor, 24 Hills Road, Cambridge CB2 1JP | August 2014

Replicates

- You should report how often each experiment was performed
- You should include a definition of biological versus technical replication
- The data obtained should be provided and sufficient information should be provided to indicate the number of independent biological and/or technical replicates
- If you encountered any outliers, you should describe how these were handled
- Criteria for exclusion/inclusion of data should be clearly stated
- High-throughput sequence data should be uploaded before submission, with a private link for reviewers provided (these are available from both GEO and ArrayExpress)

Please outline where this information can be found within the submission (e.g., sections or figure legends), or explain why this information doesn't apply to your submission:

The six replicates were generated from the MCF7 cell line with at least one passage between experiment. Therefore, each replicate is an independent biological (isogenic) replicate. No technical replicates were undertaken.

Cross-linked cells were stored at -80C until processing. All samples were processed on the same day. Samples were randomized before barcoding and pooling and then sequenced together.

While no outliers were removed replicate 5, time point 30 minutes, and replicate 6, time point 80 minutes, failed QC (very low number of reads, poor enrichment in control CTCF peaks) and were removed from the subsequent analysis.

All data is available from GEO accession number GSE119057.

Statistical reporting

- Statistical analysis methods should be described and justified
- Raw data should be presented in figures whenever informative to do so (typically when N per group is less than 10)
- For each experiment, you should identify the statistical tests used, exact values of N, definitions of center, methods of multiple test correction, and dispersion and precision measures (e.g., mean, median, SD, SEM, confidence intervals; and, for the major substantive results, a measure of effect size (e.g., Pearson's r, Cohen's d)
- Report exact p-values wherever possible alongside the summary statistics and 95% confidence intervals. These should be reported for all key questions and not only when the p-value is less than 0.05.

Please outline where this information can be found within the submission (e.g., sections or figure legends), or explain why this information doesn't apply to your submission:

Raw data for Figure 1 is displayed in Figure 1-Figure supplement 3 and in Supplementary Tables 1 and 3.

Figure 2, raw data is available in Supplementary Table 1.

Figure 3, plots for Class A and Class B are genome-wide averages for clarity (>20,000 data points), raw data is available in Supplementary Table 1. P-values quoted are generated by Homer motif analysis, citation is provided to algorithm/software.

GREAT analysis, q-values exactly as provided by the analysis tool, citation provided to method.

Figure 4A, raw data is available in Supplementary Table 1, plots are presented with error bars for clarity. Variation of data is demonstrated in Figure 1-Figure supplement 3.

Figure 4B, raw data shown.

Page 4, F-test used. P-value given in scientific notation as diminishingly small. Code for analysis provided in Rmarkdown (Rmd) as supplementary material.

(For large datasets, or papers with a very large number of statistical tests, you may upload a single table file with tests, Ns, etc., with reference to sections in the manuscript.)

eLife Sciences Publications, Ltd is a limited liability non-profit non-stock corporation incorporated in the State of Delaware, USA, with company number 5030732, and is registered in the UK with company number FC030576 and branch number BR015634 at the address 1st Floor, 24 Hills Road, Cambridge CB2 1JP | August 2014

Group allocation

- Indicate how samples were allocated into experimental groups (in the case of clinical studies, please specify allocation to treatment method); if randomization was used, please also state if restricted randomization was applied
- Indicate if masking was used during group allocation, data collection and/or data analysis

Please outline where this information can be found within the submission (e.g., sections or figure legends), or explain why this information doesn't apply to your submission:

Not relevant as experimental samples were from tissue culture.

Additional data files ("source data")

- We encourage you to upload relevant additional data files, such as numerical data that are represented as a graph in a figure, or as a summary table
- Where provided, these should be in the most useful format, and they can be uploaded as "Source data" files linked to a main figure or table
- Include model definition files including the full list of parameters used
- Include code used for data analysis (e.g., R, MatLab)
- Avoid stating that data files are "available upon request"

Please indicate the figures or tables for which source data files have been provided:

The source data for all figures has been provided and the R code is included as supplementary material.
